# The Transcription Factor Bach1 Suppresses the Developmental Angiogenesis of Zebrafish

**DOI:** 10.1155/2017/2143875

**Published:** 2017-03-14

**Authors:** Li Jiang, Meng Yin, Jie Xu, Mengping Jia, Shaoyang Sun, Xu Wang, Jianyi Zhang, Dan Meng

**Affiliations:** ^1^Department of Physiology and Pathophysiology, School of Basic Medical Sciences, Fudan University, Shanghai 200032, China; ^2^Department of Cardiothoracic Surgery, Shanghai Children's Medical Center, Shanghai Jiao Tong University School of Medicine, Shanghai 200127, China; ^3^Department of Biochemistry and Molecular Biology, School of Basic Medical Sciences, Fudan University, Shanghai 200032, China; ^4^Department of Biomedical Engineering, School of Medicine, University of Alabama at Birmingham, Birmingham, AL 35294, USA

## Abstract

Bach1 disrupts Wnt/*β*-catenin signaling, reduces the proliferation, migration, and tube formation activity of endothelial cells (ECs), and suppresses angiogenesis in mice with surgically induced hind-limb ischemia (HLI). However, the function of Bach1 during developmental angiogenesis in zebrafish remains unclear. Here, we found that zebrafish Bach1 was expressed ubiquitously during early embryonic development in zebrafish. Bach1b mRNA injection of* Tg(fli1:gfp)* fish disrupted intersegmental vessels (ISV) and dorsal longitudinal anastomotic vessels (DLAV) and suppressed endogenous Wnt/*β*-catenin signaling and Wnt8a stimulated vascular endothelial growth factor (VEGF) and interleukin-8 (IL-8) gene expression at early embryonic stages of zebrafish. Furthermore, chromatin immunoprecipitation experiments confirmed that Bach1 occupied the TCF/LEF-binding site of the VEGF promoter in human umbilical vein endothelial cells (HUVECs). Bach1 inhibited VEGF transcription by recruiting histone deacetylase 1 (HDAC1) to the VEGF promoter in HUVECs. Exogenous administration of VEGF or IL-8 partially rescued Bach1-driven antiangiogenic functions in HUVECs. Taken together, these observations indicate that Bach1 suppresses the developmental angiogenesis of zebrafish and that this function is associated with declines in Wnt/*β*-catenin signaling and VEGF and IL-8 expression.

## 1. Introduction

Wnt signaling has been implicated in cardiovascular development, including placental vascular development, endothelial cell fate specification and endothelial cell proliferation, and vascular growth and integrity [1–3]. Activation of Wnt signaling was observed during embryonic development in many types of vessels [1], and endothelial-specific deletion of *β*-catenin affected the development of the embryonic vasculature and resulted in early lethality in utero of mouse [4]. Disruption of Wnt signaling in zebrafish resulted in severe vascular defects during embryonic angiogenesis [3, 5]. Wnt signaling is also reported to promote expressions of vascular endothelial growth factor (VEGF) and interleukin-8 (IL-8) [6, 7], which have been shown to have angiogenic activity in human endothelial cells and zebrafish [8–11].

BTB and CNC homology 1 (Bach1) is a basic leucine zipper transcription factor that heterodimerizes with small Maf proteins and binds to the antioxidant responsive element (ARE) to repress the expression of genes encoding antioxidant proteins [12, 13]. We have shown that Bach1 binds to TCF4, reduces the interaction of *β*-catenin with TCF4, and disrupts Wnt/*β*-catenin signaling by recruiting histone deacetylase 1 to the promoter of TCF4-targeted genes [14]. Our results also indicate that Bach1 reduces the proliferation, migration, and tube formation activity of human endothelial cells (ECs), induces endothelial cell apoptosis and cell-cycle arrest, and suppresses angiogenesis in mice with surgically induced hind-limb ischemia (HLI) [14, 15]. These observations suggest Bach1 as a major regulator of endothelial function and ischemia-induced angiogenesis. However, little is yet known about the role of Bach1 in vascular development. Bach1 is conserved in vertebrates and some urochordates [16]. In zebrafish, two Bach1 homolog genes (Bach1a; Bach1b) have been reported and Bach1b is the main transcription factor, which heterodimerized with MafK to repress target gene expression, including heme oxygenase-1a (hmox1a) and zymogen [16–18]. Thus, in the present study, zebrafish was used as an in vivo model to study the role of Bach1b in Wnt/*β*-catenin signaling and the developmental angiogenesis, and in vitro cell transfection and chromatin immunoprecipitation (ChIP) assays were conducted to elucidate how Bach1 regulates Wnt target gene (VEGF) expression in human umbilical vein endothelial cells (HUVECs).

## 2. Methods and Materials

### 2.1. Maintenance of Zebrafish

The zebrafish AB line was used, and embryos and adult fish were raised according to the standard protocol described in* The Zebrafish Book*.* Tg(fli1:EGFP)*^y1^,* Tg(TOP:GFP)*^w25^, and* Tg(hsp701:wnt8a-GFP)*^w34^ transgenic zebrafish lines [19–21] were provided by Dr. Xu Wang (Fudan University). Embryos were obtained by natural spawning and were maintained in E3 zebrafish water at 28.5°C and staged according to Kimmel et al. [22]. Embryos were heat-shocked at the indicated stage by transferring them into egg water prewarmed to 38°C [*Tg(hsp701:wnt8a-GFP)*^w34^] and kept at this temperature for 25 minutes. After heat-shock, the plate containing the embryos was transferred back into a 28.5°C incubator. Experimental procedures and animal use and care protocols were approved by the Fudan University Animal Ethics Committee and were consistent with the “Guide for the Care and Use of Laboratory Animals” published by the National Institutes of Health (NIH) of the United States.

### 2.2. mRNA Synthesis

Zebrafish* Bach1b* gene was amplified through reverse transcription-polymerase chain reaction (RT-PCR) and verified by DNA sequencing. DNA generated by PCR can be transcribed directly from the PCR provided it contains a T7 RNA polymerase promoter upstream of the sequence to be transcribed. Then zebrafish* Bach1b* mRNAs were synthesized from linearized templates using the mMessage mMachine kit (Invitrogen, Carlsbad, CA). Zebrafish embryos were injected at the one cell stage with 200 pg of synthetic* Bach1b* mRNA in water.

### 2.3. Whole Mount Antibody Staining

Zebrafish embryos were fixed two hours in 4% paraformaldehyde (PFA)/phosphate-buffered saline (PBS), washed, dehydrated in methanol, and stored at −20°C. Embryos were rehydrated, permeabilized with proteinase K, and fixed again with 4% PFA/PBS. After blocking in 1% bovine serum albumin (BSA) plus 2% serum in PBS, embryos were incubated with an anti-Bach1 antibody (ab115210, Abcam, Cambridge, MA) at 4°C overnight. On the following day embryos were washed and the secondary antibody was added at 4°C overnight. The color reaction was developed with the avidin–biotin–horseradish peroxidase method (ABC kit) and diaminobenzidine (DAB) as a chromogen.

### 2.4. Quantitative Real-Time Reverse Transcription-Polymerase Chain Reaction

The expression levels of various genes in zebrafish embryos or HUVECs were analyzed by real-time RT-PCR as described previously [23]. Eight to ten zebrafish embryos were collected. Total RNA was extracted using the TRIzol reagent (Invitrogen, Carlsbad, CA) according to the manufacturer's instructions. cDNA was synthesized using SuperScript Reverse Transcriptase (Fermentas, Glen Burnie, MD). RT-PCR analysis was carried out using SYBR Green PCR master mix (Toyobo, Japan). Primers are shown in Table 1. All samples were analyzed using a Bio-Rad real-time analyzer (Bio-Rad Laboratories, Hercules, CA) and results were normalized to the glyceraldehyde-3-phosphate dehydrogenase (GAPDH) expression. Experiments were done at least in triplicate.

### 2.5. Confocal Microscopy and Imaging

Zebrafish embryos were examined by confocal microscopy; the embryos were embedded live in 1% low-melting-point agarose (Sigma-Aldrich, St. Louis, MO) and photographed by a Leica SP8 confocal microscopy system (Bio-Rad, Hercules, CA). Other fluorescent images were taken under Olympus MVX10 (Olympus Corporation) and the fluorescence intensity was quantified using ImageJ software (National Institutes of Health, Bethesda, MA).

### 2.6. Cell Culture and Tube Formation Assay

Primary human umbilical vein endothelial cells (HUVECs) were obtained from fresh umbilical cord veins from normal pregnancies, and informed consent was received from the pregnant women; a procedure that was approved by the ethics review board of Fudan University. HUVECs were isolated by collagenase digestion and identified as previously described [14]. The recombinant adenoviruses encoding human Bach1 gene (AdGFP-Bach1) or GFP Control (AdGFP) were purchased from Genechem (Shanghai, China) and used to infect the ECs. For the transduction, a multiplicity of infection (MOI) of 25 was used. No detectable cellular toxicity was observed. HUVECs were cultured in presence of VEGF-A (50 ng/mL, PeproTech Inc, Rocky Hill, NJ) or IL-8 (50 ng/mL, PeproTech Inc, Rocky Hill, NJ) in a 24-well plate coated with 200 *μ*L of growth factor-reduced Matrigel. Tube length was quantified after 12 hours by measuring the cumulative tube length in five random microscopic fields with an inverted phase contrast microscope using ImageJ software (National Institutes of Health, Bethesda, MA). The relative tube lengths were expressed as the fold change relative to the Control. Each experiment was repeated four independent times.

### 2.7. Transient Transfection and Luciferase Assay

Transient transfection and luciferase assay were performed as described previously [24]. A 2.65 kb human VEGF gene promoter was kindly provided by Dr. Jiang (Thomas Jefferson University, USA). VEGF promoter fragments of different sizes (−529 and −228) were amplified from human genomic DNA with appropriate sets of primers. PCR products were cloned into the luciferase vector pGL3-basic. All constructs were verified by sequencing. Cells were transfected with DNA or siRNA together with the indicated VEGF reporter or pGL3-basic luciferase reporter and *β*-galactosidase plasmid using Lipofectamine 2000. The transfected cells were cultured for 24 hours. Trichostatin A (TSA, T1952, Sigma-Aldrich, St. Louis, MO) was added for 24 hours. Cells were harvested and measured with a luciferase assay kit (Promega, Madison, WI). Relative luciferase activity was calculated as the ratio of Luc/*β*-galactosidase activity and expressed as the ratio of VEGF reporter/pGL3-basic. Each experiment was repeated three independent times.

### 2.8. Chromatin Immunoprecipitation (ChIP)

The assay was performed according to the manufacturer's protocol (ChIP-IT protein G magnetic beads kit, Active Motif, Carlsbad, CA). In brief, cells were seeded onto a 10 cm culture dish and one day later were cross-linked by 1% formaldehyde for 10 minutes at room temperature and followed by fragmentation of genomic DNA using a sonification apparatus. Cell lysates were used for chromatin immunoprecipitation using Bach1 (sc-14700, Santa Cruz, CA), HDAC1 (sc-7872, Santa Cruz, CA), Ac-Histone H3 (sc-56616, Santa Cruz, CA), and Control antibody (sc-2028, Santa Cruz, CA). The protein G magnetic beads-antibody/chromatin complexes were washed and the antibody/chromatin complexes were subsequently eluted. The cross-linked protein/DNA complexes were detached at 65°C for four hours followed by purification of the genomic DNA. PCR primers amplifying human VEGF promoters are shown in Table 1. Real-time PCR was carried out using SYBR Green PCR master mix. The amount of DNA associated with each protein (relative to the total amount of DNA used) was determined.

### 2.9. Western Blotting

Western blotting was performed as described previously [25]. Briefly, cells or tissue homogenates were lysed with SDS sample buffer on ice for 10 minutes. Samples were heated at 95°C for five minutes. SDS-PAGE was performed and proteins were detected using antibodies to Bach1 (sc-14700, Santa Cruz, CA) and *β*-actin (A5316, Sigma-Aldrich, St. Louis, MO). Chemiluminescence was detected on a Tanon-5500 Imaging System (Tanon Science & Technology Ltd, Shanghai, China).

### 2.10. Measurement of Secreted VEGF via ELISA

Human VEGF-A was quantified in the cell culture supernatant using ELISA assay kit from R&D Systems, Inc. (Minneapolis, MN) as described previously [23]. Briefly, ECs were transfected with siRNA or transduced with adenovirus. After 24 hours, cells were washed with phosphate-buffered saline (PBS) and fresh medium was added. Supernatants of the cells were collected at 48 hours and the number of cells was counted. An ELISA assay was performed according to the manufacturer's protocol. After measuring the VEGF-A content in the supernatants, the amount of VEGF-A per 10^6^ cells was calculated. Each experiment was repeated three independent times.

### 2.11. Statistical Analysis

Data are expressed as mean ± SEM from in the Figures. Differences among groups were determined using Prism software with one-way analysis of variance followed by Bonferroni post hoc test. Differences between two groups were assayed by two-tailed Student's *t*-test. The 0.05 level of probability was used as the criterion of significance.

## 3. Results

### 3.1. Bach1 Was Ubiquitously Expressed during Early Embryonic Development in Zebrafish

**Figure 1:**
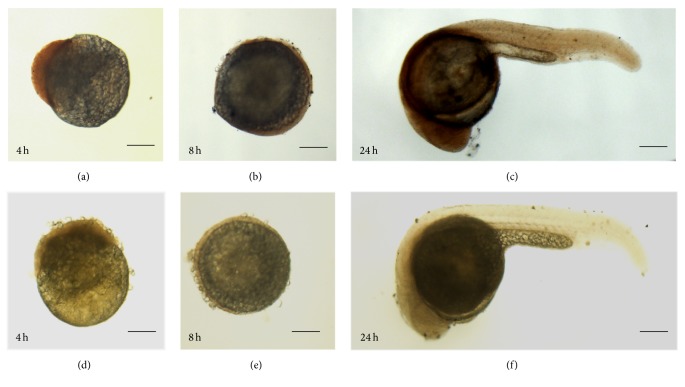
The expression of Bach1 during embryonic development in zebrafish. Bach1 expression was evaluated in zebrafish embryos that had been stained with (a–c) or without (d–f) a Bach1 antibody at 4 h (a, d), 8 h (b, e), and 24 h (c, f) after fertilization. Scale bar: 100 *μ*m.

We observed that zebrafish Bach1 expression was evident as early as 4 hpf (hours after fertilization) and maintained up to 24 hpf and ubiquitously expressed during early embryonic zebrafish development (Figure 1).

### 3.2. Overexpression of Bach1b Impairs the Developmental Angiogenesis of Zebrafish

**Figure 2:**
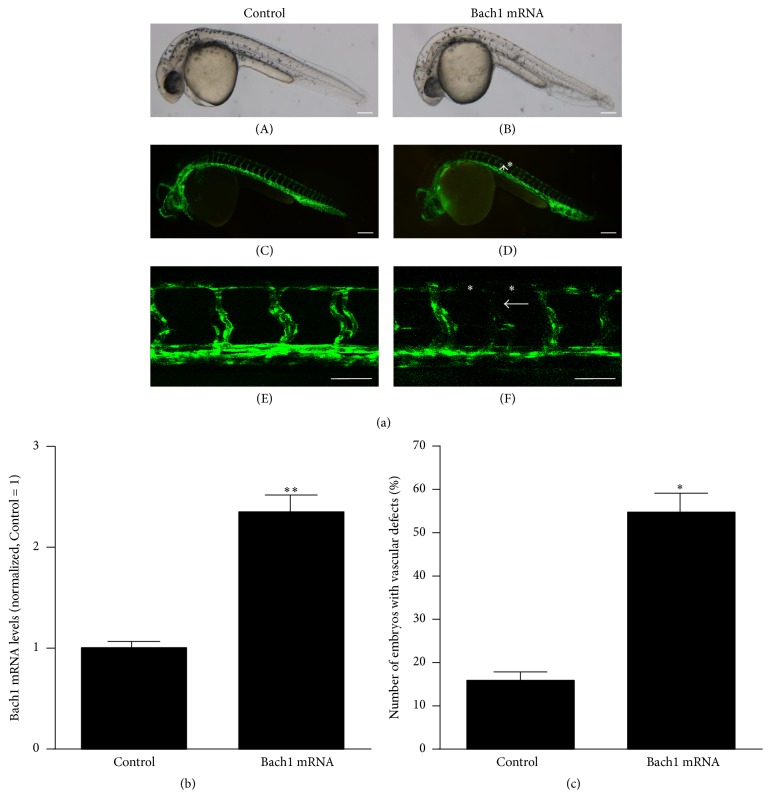
Bach1 overexpression induces vascular defects in zebrafish embryos. (a) Zebrafish vasculature was evaluated via fluorescent images (C-D) or confocal images (E and F) in normal 32-hpf* Tg(fli1:EGFP)* zebrafish embryos (A, C and E) and in embryos that had been injected with 200 pg of Bach1 mRNA (B, D and F) at the single-cell stage. Bach1 mRNA injections disrupted formation of the intersegmental vessels (ISV) (arrow) and the dorsal longitudinal anastomotic vessels (DLAV) (asterisks). (A–D) Scale bar: 100 *μ*m. (E and F) Scale bar: 50 *μ*m. (b) Bach1 mRNA levels were evaluated in normal and Bach1-mRNA–injected embryos via quantitative real-time RT-PCR (*n* = 8; ^*∗∗*^*P* < 0.01 versus Control). (c) Embryos with defects in ISV and DLAV were counted and expressed as a percentage of the total number of embryos in each experimental group (*n* = 50; ^*∗*^*P* < 0.05 versus Control).

Bach1b plays an important role in Nrf2-MafK pathway in zebrafish [17]. Thus, we overexpressed Bach1b in zebrafish embryos to investigate the role of Bach1 during vascular development. Injection of Bach1b mRNA into the one cell stage of* Tg(fli1:EGFP)* fish embryos resulted in ≈2-fold increase in mRNA expression of Bach1b at 32 hpf as evidenced by qRT-PCR (Figure 2(b)). No detectable apoptosis and toxicity were observed after injection of Bach1b mRNA with 200 pg for each embryo (data not shown). The analysis of the vasculature revealed that intersegmental vessels (ISV) and the dorsal longitudinal anastomotic vessels (DLAV) had not formed properly in Bach1b-overexpressing zebrafish (Figures 2(a) and 2(c)). These results suggest that Bach1b overexpression impairs the developmental angiogenesis of zebrafish embryos.

### 3.3. Bach1 Suppresses Endogenous Wnt/*β*-Catenin Signaling and Exogenous Wnt8a Stimulated VEGF and IL-8 Gene Expression in Zebrafish

**Figure 3:**
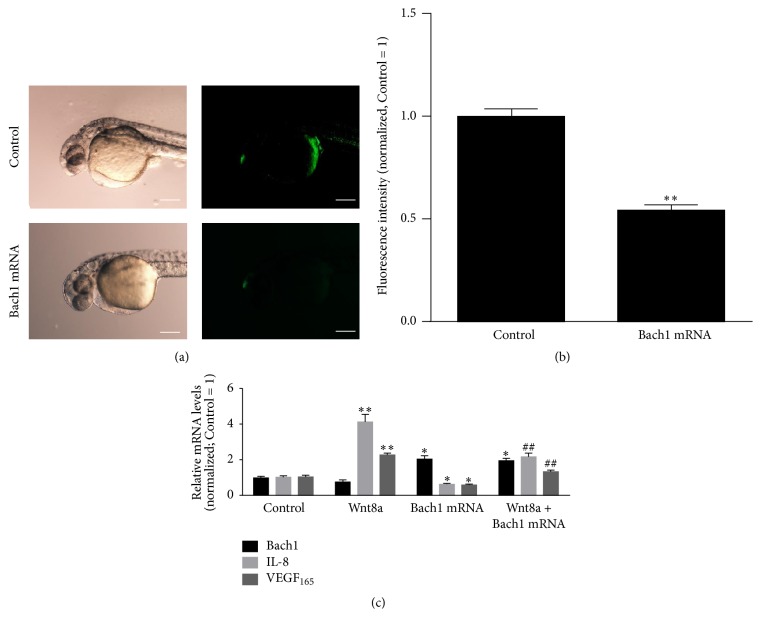
Bach1 suppresses endogenous Wnt/*β*-catenin signaling and exogenous Wnt8a stimulated VEGF and IL-8 gene expression in zebrafish. (a) Representative fluorescent images of 36-hpf* Tg(TOP:GFP)* transgenic zebrafish embryos that had been injected with or without Bach1 mRNA. (b) The fluorescence intensity of GFP was quantified and expressed as the as the ratio of measurements Bach1 mRNA and Control group (*n* = 12; ^*∗∗*^*P* < 0.01 versus Control). (c) IL-8, VEGF_165_, and Bach1 mRNA levels were evaluated in 32-hpf* Tg(hsp:wnt8a)* embryos following Bach1 mRNA injections at the one cell stage and Wnt pathway activation (heat-shock) at 24 hpf (*n* = 10; ^*∗*^*P* < 0.05, ^*∗∗*^*P* < 0.01 versus Control; ^##^*P* < 0.01 versus Wnt8a).

Recently, we have shown that Bach1 disrupts Wnt/*β*-catenin signaling in HUVECs [14]. We then investigated whether Bach1b inhibits Wnt/*β*-catenin signaling at early embryonic stages of zebrafish. We made use of the* Tg(TOP:GFP)* transgenic line of zebrafish in which *β*-catenin dependent transcriptional activity can be monitored by expression of a GFP reporter driven from a promoter containing multiple TCF/LEF elements [20]. Analysis of TOP:GFP expression showed that a significant decrease in fluorescence intensity with GFP at 36 hours after injection of Bach1b mRNA (Figures 3(a) and 3(b)), indicating that Bach1b suppresses endogenous Wnt/*β*-catenin activity in zebrafish. We then used heat-shock line (*hsp:wnt8a*) to activate Wnt/*β*-catenin signaling pathway. Heat-shock alone did not cause significant apoptosis in zebrafish. The mRNA expression of Wnt target genes (VEGF_165_ and IL-8/CXCL8) was increased when Wnt signaling was activated. However, the effect of Wnt8a stimulation was partially abolished by higher levels of Bach1 expression in zebrafish (Figure 3(c)).

### 3.4. Bach1 Occupies the TCF/LEF-Binding Site of the VEGF Promoter and Recruits HDAC1 to the VEGF Promoter and Then Inhibits VEGF Expression in HUVECs

**Figure 4:**
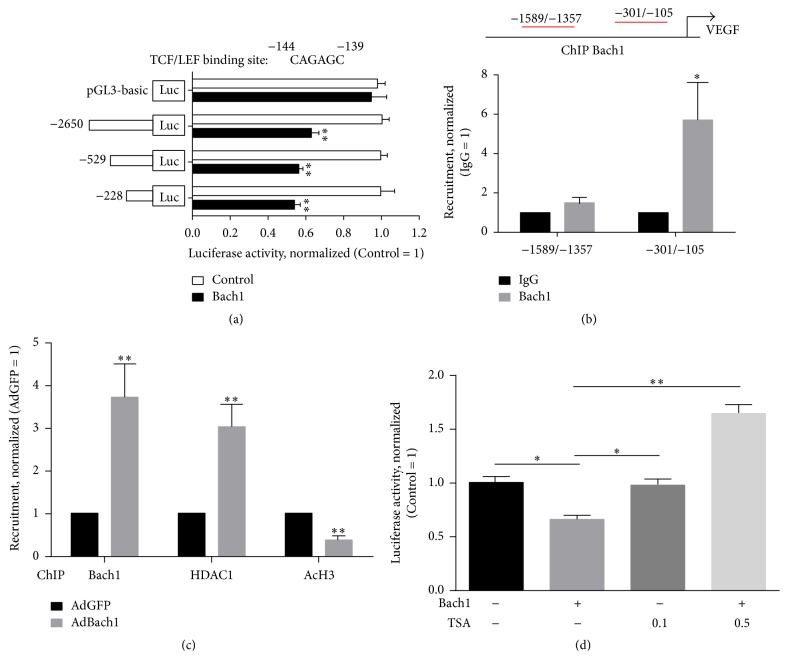
Bach1 occupies the TCF/LEF-binding site of the VEGF promoter and recruits HDAC1 to the VEGF promoter in HUVECs. (a) Potential TCF/LEF-binding site of the VEGF promoter is shown. Luciferase reporter constructs were created containing the truncated (−2650, −529, and −228) versions of the VEGF promoter; then the VEGF reporter or a pGL3-basic luciferase reporter and *β*-gal were cotransfected with a Bach1-coding vector or with an empty vector (Control) into HEK293T cells, and luciferase activity was evaluated 48 hours later (*n* = 3; ^*∗∗*^*P* < 0.01 versus Control). (b) Chromatin immunoprecipitation DNA was isolated with an anti-Bach1 antibody or with a Control IgG antibody, and then quantitative real-time RT-PCR analyses were performed with primer sequences from the indicated regions of the VEGF promoter in HUVECs (*n* = 4; ^*∗*^*P* < 0.05 versus Control IgG). (c) HUVECs were transfected with AdGFP or AdBach1; then chromatin immunoprecipitation was performed with antibodies against the indicated proteins. Quantitative real-time RT-PCR analyses were performed with a primer for the VEGF promoter (−301/−105) (*n* = 3; ^*∗∗*^*P* < 0.01 versus AdGFP). (d) HUVECs were transfected with AdGFP or AdBach1 and with the VEGF (−228) promoter construct; then, the cells were treated with or without TSA (0.1 or 0.5 *μ*mol/L) for 24 hours and luciferase activity was quantified (*n* = 3; ^*∗*^*P* < 0.05, ^*∗∗*^*P* < 0.01).

**Figure 5:**
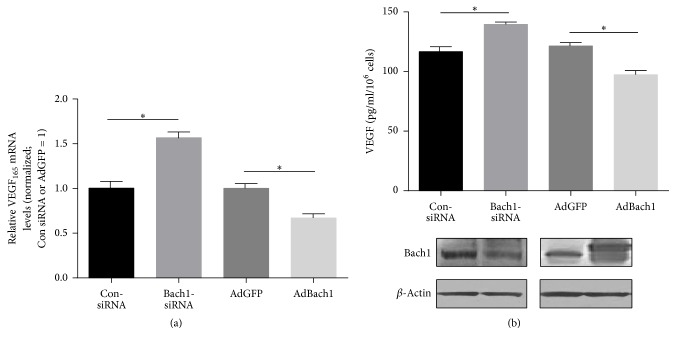
Bach1 inhibits VEGF mRNA and protein expression in HUVECs. (a and b) mRNA or protein levels of VEGF_165_ were compared in ConsiRNA- and Bach1siRNA-transfected HUVECs and in AdGFP and AdBach1-HUVECs. mRNA levels (a) were evaluated via quantitative PCR (*n* = 4; ^*∗*^*P* < 0.05) and protein levels (b) were evaluated via ELISA (*n* = 3; ^*∗*^*P* < 0.05).

We previously demonstrated that Bach1 interacted with TCF4 and occupied the TCF4 binding site of the IL-8 promoter and then repressed IL-8 expression in HUVECs [14]. To further validate VEGF as a Bach1 downstream target, we performed VEGF reporter assays using different VEGF reporter constructs and then investigated the effect of Bach1 overexpression on the luciferase activity of these constructs. In HEK293T cells, Bach1 overexpression significantly reduced the luciferase activity of all reporter constructs that contained the TCF/LEF-binding site (i.e., −2650, −529, and −228) (Figure 4(a)). The results from chromatin immunoprecipitation assays indicated that Bach1 bound to the region from position −105 to −301 of the VEGF promoter in HUVECs (Figure 4(b)), which contains the consensus TCF/LEF-binding site (positions −144 to −139, Figure 4(a)), further confirming that Bach1 occupies the TCF/LEF-binding site of the VEGF promoter. We have recently shown that Bach1 interacts with histone deacetylase 1 (HDAC1) and recruits HDAC1 to the IL-8 Promoter in HUVECs [14]. We then asked whether the inhibitory effect of Bach1 on VEGF expression is related to HDAC1. Chromatin immunoprecipitation assays indicated that HDAC1's occupancy of the VEGF promoter was ≈3-fold greater in AdBach1-HUVECs than in AdGFP-HUVECs, whereas the enrichment of H3 acetylation at these binding sites was decreased in AdBach1-HUVECs (Figure 4(c)). Furthermore, the HDAC inhibitor Trichostatin A (TSA) treatment increased the transcriptional activity of the VEGF promoter in Bach1-overexpressing HUVECs (Figure 4(d)), indicating that Bach1 may repress VEGF gene transcription by recruiting HDAC1. In addition, the results from our cellular experiments also indicated that higher levels of Bach1 expression reduced VEGF mRNA and protein levels, whereas knockdown of Bach1 expression increased VEGF mRNA and protein expression in HUVECs (Figures 5(a) and 5(b)).

### 3.5. Exogenous Administration of VEGF or IL-8 Partially Rescued Bach1-Driven Antiangiogenic Response in HUVECs

**Figure 6:**
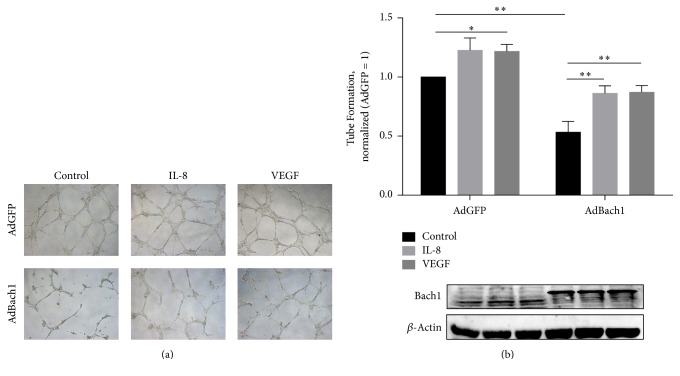
Exogenous administration of VEGF or IL-8 partially rescued Bach1-driven antiangiogenic response in HUVECs. (a) HUVECs were transfected with AdGFP or AdBach1 for 24 hours and then incubated with or without VEGF (50 ng/mL) or IL-8 (50 ng/mL) for 24 hours. Endothelial tube formation assay in Matrigel was performed in presence of VEGF or IL-8. Tube length was quantified and expressed as the fold change relative to AdGFP (*n* = 4; ^*∗*^*P* < 0.05, ^*∗∗*^*P* < 0.01). Scale bar: 500 *μ*m. (b) Bach1 and *β*-actin protein levels were evaluated via Western blot.

To determine the role of VEGF and IL-8 in the regulation of angiogenesis by Bach1, we performed Matrigel tube-forming assays in Bach1 expressing HUVECs with external VEGF or IL-8 administration. Exogenous administration of VEGF or IL-8 partially rescued Bach1-driven antiangiogenic response in HUVECs (Figure 6).

## 4. Discussion

We have shown that Bach1 is implicated in ischemia-induced angiogenesis in mice [14]. However, the function of Bach1 in developmental angiogenesis is not well understood. Here, we found that Bach1 suppressed the developmental angiogenesis of zebrafish and that this function was associated with declines in Wnt/*β*-catenin signaling and VEGF and IL-8 expression in zebrafish. Thus, Bach1 functions not only in angiogenesis after ischemic injury but also in development angiogenesis.

In the zebrafish genome, there are two Bach1 homolog genes, bach1a and Bach1b, which are located on chromosomes 15 and 10, respectively [17]. Bach1b contains both BTB and BZIP domains and mediates the regulatory role of heme in transcription of the zymogens in zebrafish [17, 18]. Recent study has identified that Bach1b is expressed ubiquitously from one cell to 48 hpf [18], and this observation is consistent with our observation. In zebrafish, the sprouting and extension of endothelial cells to form intersegmental vessels (ISV) from the dorsal aorta is considered as angiogenesis [26]. In Bach1b overexpression zebrafish, we found that the extension of ISV was dramatically hindered, suggesting that Bach1b plays a key role in the proliferation and migration of ECs during sprouting angiogenesis. In fact, our previous study has shown that Bach1 suppresses endothelial cell migration, proliferation, and tube formation in HUVECs [14]. VEGF-A is expressed in the ventromedial region of each somite during sprouting angiogenesis [10, 27]. Similar to human, VEGF121 and VEGF165 are the two most abundant isoforms of VEGF-A in the zebrafish. Zebrafish express two VEGFR2 homologs known as Kdrl/Flk1 and Kdr/Kdrb. VEGF-A/VEGFR2 signaling is required for early intersegmental vasculature development with mutation in VEGF-aa or loss of VEGFR2 homologs causing nearly complete inhibition of the formation of the ISV [26]. VEGF-A signaling has been shown to direct endothelial cells to migrate out of existing vasculature [28], and impaired endothelial migration was associated with abnormal formation of the ISV [29]. IL-8 is another key proangiogenic factor. IL-8 and its receptors CXCR1 and CXCR2 have been described in zebrafish [30], and silencing of IL-8 expression in zebrafish inhibited vascular development [11]. The results presented here indicate that Bach1b overexpression in zebrafish reduced VEGF_165_ and IL-8 mRNA levels and exogenous VEGF-A or IL-8 was able to partially reverse Bach1-driven antiangiogenic response in HUVECs, confirming VEGF and IL-8 as the major targets of Bach1-mediated antiangiogenic functions. Thus, Bach1 appears to impair the developmental angiogenesis in zebrafish by reducing the expression of VEGF and IL-8 and by impairment of endothelial cell migration and proliferation.

The Wnt/*β*-catenin signaling pathway plays a vital role in vascular development and the process of angiogenesis [31], and the inhibition of *β*-catenin has been shown to suppress angiogenesis in zebrafish [32]. These observations are consistent with our results. We found that Bach1 suppressed endogenous Wnt signaling in zebrafish, and this decline was associated with the decrease in the developmental angiogenesis of zebrafish embryos. Wnt target genes including VEGF and IL-8 are transcriptionally activated by *β*-catenin/TCF signaling in HUVECs [7, 31]. The gene promoter of human VEGF-A contains several consensus binding sites for *β*-catenin/TCF [33]. We have shown that Bach1 binds directly to TCF4 and reduces the interaction of *β*-catenin with TCF4 and then impairs Wnt/*β*-catenin signaling in HUVECs [14]. However, the mechanisms underlying the regulation of VEGF by Bach1 are mostly unknown. Our results indicated that Bach1 occupies the TCF/LEF-binding site of the human VEGF promoter, and Bach1 suppresses VEGF transcription by, at least in part, recruiting HDAC1 to the VEGF promoter. Similar results were also obtained in the transcriptional regulation of IL-8 by Bach1 [14]. Previous investigation has shown that Bach1 inhibits oxidative stress-induced cellular senescence by recruiting HDAC1 to the promoter of a subset of p53-targeted genes [34]. Thus, HDAC1 may be a key component of the mechanism(s) by which Bach1 impedes gene transcription.

Bach1 has previously been shown to inhibit Nrf2-dependent induction of heme oxygenase-1 (HO-1) gene expression [13, 35]. Our previous study also indicates that HO-1 was repressed by Bach1 during arsenite-mediated angiogenesis [36]. HO-1 has been shown to promote angiogenesis in ischemic hearts by induction of VEGF [37]. Thus, Bach1 likely inhibits angiogenesis by regulating a variety of factors, including VEGF, IL-8, and HO-1. In addition, the results from our recent investigation suggest that higher levels (≈100-fold increase in mRNA expression) of Bach1 enhanced ROS production and induced apoptosis in ischemic mouse hindlimbs [15]. However, we found that a slight (≈2-fold) increase in Bach1b mRNA expression did not induce the apoptosis (data not shown) in zebrafish in the present study, which suggests that the effect of Bach1b overexpression on the developmental angiogenesis in zebrafish cannot be explained by apoptosis.

In conclusion, the results presented here indicate that Bach1 suppresses the developmental angiogenesis of zebrafish and that this function is associated with declines in Wnt/*β*-catenin signaling and VEGF and IL-8 expression. Bach1 inhibits VEGF transcription by recruiting HDAC1 to the VEGF promoter in HUVECs. Thus, the identification of Bach1's role in developmental angiogenesis may provide new insight into angiogenesis-based therapeutic approaches.

## Figures and Tables

**Table 1:** Primers used for qPCR and ChIP.

Primer	Sequence (5′-3′)
Bach1b-F (zebrafish)	CTTGCTGGAGTTCGCCTACA
Bach1b-R (zebrafish)	AATGTACCTCTGTTGCTGTCA
IL-8-F (zebrafish)	AAGCCGACGCATTGGAAAAC
IL-8-R (zebrafish)	AGGGGTCCAGACAGATCTCC
VEGF_165_-F (zebrafish)	ATCGAGCACACGTACATCCC
VEGF_165_-R (zebrafish)	CCTTTGGCCTGCATTCACAC
GAPDH-F (zebrafish)	GAGGCTTCTCACAAACGAGGA
GAPDH-R (zebrafish)	TGGCCACGATCTCCACTTTC
Human VEGF ChIP (−301/−105)-F	CACTTTCCTGCTCCCTCCTC
Human VEGF ChIP (−301/−105)-R	AGCCTCAGCCCTTCCACA
Human VEGF ChIP (−1589/−1357)-F	GAGGCTATGCCAGCTGTAGG
Human VEGF ChIP (−1589/−1357)-R	CCCTTTTCCTCCAACTCTCC
Human VEGF_165_-F	ATCTTCAAGCCATCCTGTGTGC
Human VEGF_165_-R	CAAGGCCCACAGGGATTTTC
Human GAPDH-F	CCATCTTCCAGGAGCGAGATC
Human GAPDH-R	GCCTTCTCCATGGTGGTGAA
