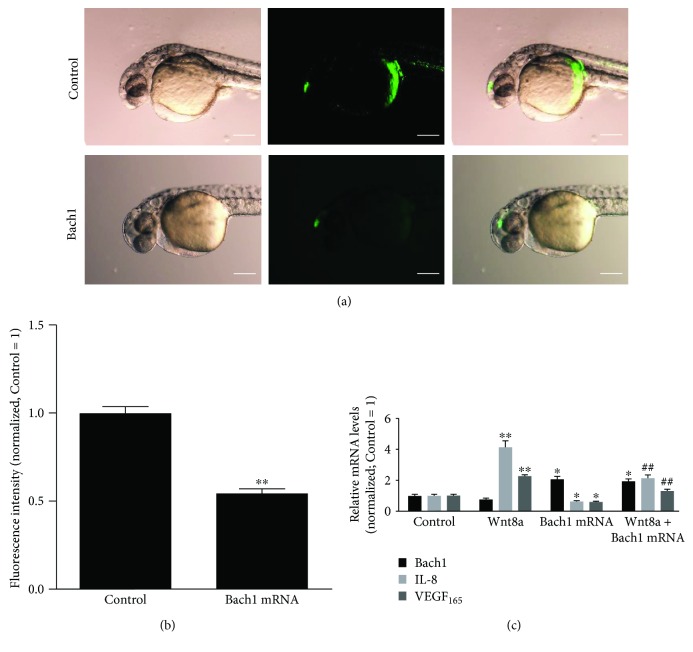# Corrigendum to “The Transcription Factor Bach1 Suppresses the Developmental Angiogenesis of Zebrafish”

**DOI:** 10.1155/2018/2852343

**Published:** 2018-10-25

**Authors:** Li Jiang, Meng Yin, Jie Xu, Mengping Jia, Shaoyang Sun, Xu Wang, Jianyi Zhang, Dan Meng

**Affiliations:** ^1^Department of Physiology and Pathophysiology, School of Basic Medical Sciences, Fudan University, Shanghai 200032, China; ^2^Department of Cardiothoracic Surgery, Shanghai Children's Medical Center, Shanghai Jiao Tong University School of Medicine, Shanghai 200127, China; ^3^Department of Biochemistry and Molecular Biology, School of Basic Medical Sciences, Fudan University, Shanghai 200032, China; ^4^Department of Biomedical Engineering, School of Medicine, University of Alabama at Birmingham, Birmingham, AL 35294, USA

In the article titled “The Transcription Factor Bach1 Suppresses the Developmental Angiogenesis of Zebrafish” [1], the quality of the images for Figures 2(a) and 3(a) is low. Higher-quality images for Figures 2(a) and 3(a) and merged image of bright field and fluorescent image for Figure 3(a) are shown as follows.

## Figures and Tables

**Figure 1:**
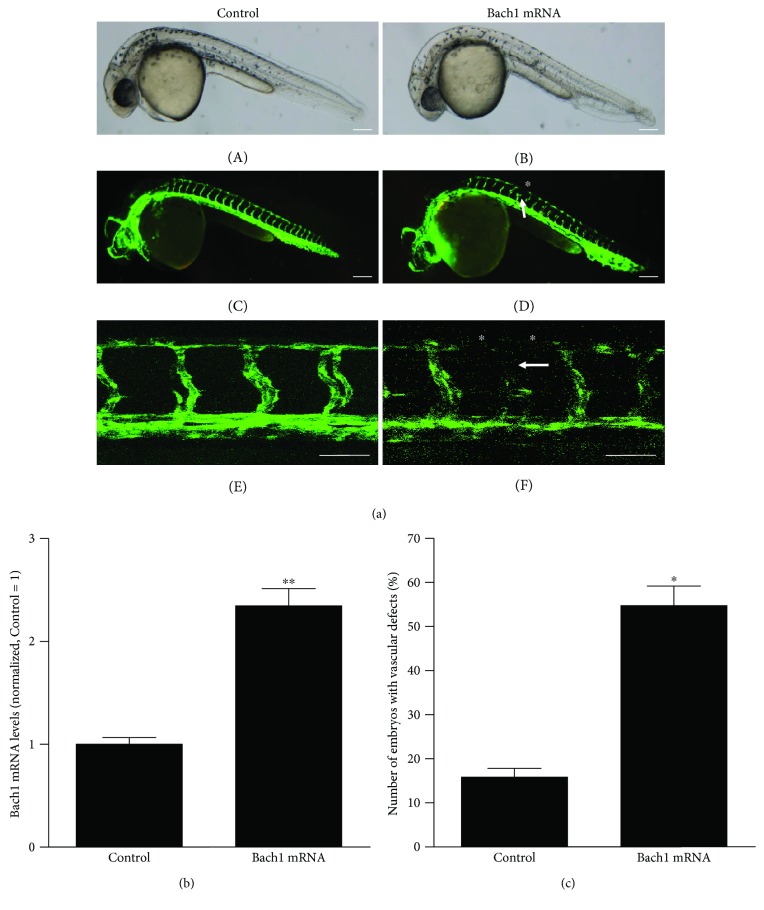


**Figure 2:**